# Nutritional Evaluation of Milk Thistle Meal as a Protein Feedstuff for Diets of Dairy Cattle

**DOI:** 10.3390/ani14131864

**Published:** 2024-06-24

**Authors:** Modinat Tolani Lambo, Rui Liu, Xianglong Zhang, Yonggen Zhang, Yang Li, Manji Sun

**Affiliations:** College of Animal Science and Technology, Northeast Agricultural University, Harbin 150030, China; motolanilambo@neau.edu.cn (M.T.L.); lr110916@163.com (R.L.); zhangxianglong0908@163.com (X.Z.); zhangyonggen@neau.edu.cn (Y.Z.)

**Keywords:** milk thistle meal, nutritional value, Fourier transform infrared technology, soybean meal, dairy cattle, CNCPS

## Abstract

**Simple Summary:**

This study aimed to assess the feeding value of milk thistle meal for dairy cattle production by comparing it with seven conventional protein feed resources. The nutritional evaluation revealed that milk thistle meal had a higher fiber content and non-degradable carbohydrate levels compared to most other feedstuffs. Despite its poor carbohydrate composition, it exhibited a higher crude protein rumen effective degradation rate, similar to soybean meal, and had a valuable source of methionine, an essential amino acid. However, it showed lower small intestinal rumen undegradable protein digestibility. The study highlights the potential of milk thistle meal as a nutrient-rich feed supplement for dairy cattle, suggesting avenues for further improvement to enhance its application in dairy feeding practices.

**Abstract:**

The objective of this work is to investigate the chemical and nutritional value of milk thistle meal (MTM) in order to improve it and to provide theoretical support for its application in dairy cattle production. MTM was assessed in comparison with seven conventional protein feed sources, namely, soybean meal (SBM), cottonseed meal (CS), canola meal (CN), palm kernel meal (PK), rice bran meal (RB), corn germ meal (CG), and sesame meal (SS). The chemical composition of these feedstuffs was assessed using wet chemical analysis, the Cornell Net Carbohydrate and Protein System was used to evaluate the carbohydrate and protein fractions, and the in situ nylon bag technique and the modified three-step in vitro method were used to assess the rumen degradation and intestinal digestibility. Additionally, Fourier transform infrared technology was used to determine the feedstuff protein spectral molecular structure and its amino acid profile was also assessed. The result showed that MTM acid detergent fiber, lignin, unavailable nitrogen, and non-degradable carbohydrate content were higher than those of the other feedstuffs. It had a 17% and 36% rumen effective degradation rate of neutral detergent fiber and dry matter, respectively, and had the lowest small intestinal rumen undegradable protein digestibility rate. It was low in leucine, histidine, arginine, and proline, but high in methionine. The total area of amide I and amide II in the protein secondary structure was similar to that of CN and CS, and the amide I and II ratio was not different from that of RB. To sum up, MTM has a poor carbohydrate composition and is high in fiber but, in comparison to most other protein feeds, has a higher crude protein rumen effective degradation rate, similar to that of SBM, and it is a good source of methionine, a limiting amino acid. Hence, its nutritional value can be further improved for application in dairy feeding through processes such as microbial or enzymatic fermentation.

## 1. Introduction

The livestock feed industry continues to face a global challenge of feed resource shortage due to the rapid expansion in animal production coupled with the growing human population, resulting in severe competition for human-edible feed resources and culminating in an increasing rise in the prices of conventional animal feed ingredients. This impacts the animal industry severely and necessitates the need to explore alternative, less expensive, locally available and unconventional feed resources, i.e., feed ingredients not commonly utilized in feed composition [1]. Studies have shown that protein remains a major and critical nutritional limiting factor for ruminants [2]. However, feedstuffs with high-quality protein sources like soybean meal and canola meal may be difficult to replace, due to their balanced amino acid (AA) ratio and rich nutrient composition. Milk thistle (*Silybum marianum*) belongs to the family *Asteraceae* and is an annual/biennial plant with high economic value and prospects. Milk thistle contains a considerable amount of protein, fat, flavonolignans, and linoleic and oleic acids [3,4]. It is known for its richness in bioactive compounds, such as total phenolics, flavonoids, tannins, tocopherol, and silymarin, a flavonolignan, which is its most abundant bioactive compound [5,6], hence the increasing interest in its use in animal feeding. Studies have reported the benefits of utilizing various milk thistle byproducts, such as its oil, fruits, and seeds, in the feed of different livestock species. It was reported that feeding 2% milk thistle seed powder enhanced the carcass characteristics and the physical and chemical characteristics of lamb, *Longissimus thoracic*, and also improved its growth performance [7]. Supplementing the fattener diet of pigs with milk thistle seed increased the body weight gain and enhanced meat color, texture, water-holding capacity, and oxidative capacity, while it increased polyunsaturated fatty acid (PUFA) [8]. Karaiskou et al. [9] also observed that supplementing milk thistle oil in ewe’s diet translated into higher daily milk yield and enhanced its nutrient value by increasing its composition of monounsaturated fatty acids and PUFA and lowering saturated fatty acid content. 

Milk thistle meal (MTM) is a byproduct of milk thistle seed oil extraction [10], and it is gaining attention in the animal industry. Milk thistle seed is made up of up to 26% oil [11], and, after extraction, there remains a residual of silymarin [12], which has various biological and functional properties, such as improving lipid metabolism, inflammation, hepato-protection, and anti-oxidation [13,14], and could be a prospective alternative plant protein feedstuff. Using an alternative feedstuff like MTM is beneficial to alleviate the scarcity of feed resources and lower feed prices; however, unconventional feed sometimes has the general drawback of poor nutrient compositions such as low protein content or AA imbalance and possibly high antinutrients [15,16]. Halmemies-Beauchet-Filleau et al. [17] stated that the possibility of adopting new and unconventional feedstuffs for ruminants relies on the feeding value of such feed ingredients, the response of the animal, its impact on productivity, and its economic value in comparison to its conventional counterpart. According to Hashemi Jabali et al. [18], MTM has a protein content of about 25–30%, and its neutral detergent fiber (NDF) could also be as high as 580 g/kg, which often limits its utilization in ruminants or even results in it being discarded. In addition, it is speculated that it has an insufficient limiting AA content. Currently, information is scarce on the nutritional value, digestibility, and degradation characteristics of MTM, which will limit the possibility of developing it by improving its feeding value and maximizing its potential for application in ruminants.

The Cornell Net Carbohydrate and Protein System (CNCPS) is an appropriate nutritional model that can easily and accurately assess the available nutrient resources in feeds [19] and reflect its degradation rate in the rumen, its digestibility, and its nutrient absorption efficiency. The CNCPS model can also be used to assess the nutrient value of the carbohydrate and protein components of the feed resource [20]. The in situ nylon bag technique can predict the actual rumen degradation and digestion of the nutrients of prospective feedstuff and the feeding value of feeds for livestock [21]. Furthermore, with regards to protein feedstuff utilization in ruminants, the quality and nutritional value, degradability and digestibility, availability, and metabolism of protein do not depend solely on the crude protein and AA composition but are also dependent on the feeds’ protein intrinsic molecular structures [22]. The adoption of molecular spectroscopy, such as Fourier transform infrared (FTIR) technology, provides a fast, simple, highly sensitive, and non-invasive analysis of the protein quality of various feed samples by evaluating the primary and secondary protein structures with high resolution. The nutrient value of a protein feed can be predicted by the height and area of the amide I and II bands and the α-helix peak [23]. 

Therefore, this research was carried out to compensate for the gap in information on the feeding value of milk thistle meal for potential utilization as an alternative feed ingredient by comparing it with other conventional protein feedstuffs. The study compared milk thistle meal with seven other conventional protein feedstuffs based on their chemical composition, rumen degradation, small intestinal digestibility, carbohydrate and protein sub-fractions, amino acid profile, and protein secondary structure using the wet chemical method, the in situ nylon bag technique, the modified three-step in vitro procedure, CNCPS, and FTIR spectroscopy.

## 2. Materials and Methods

### 2.1. Feed Sampling and Chemical Composition

Soybean meal (SBM), canola meal (CNM), cottonseed meal (CSM), palm kernel meal (PKM), rice bran meal (RBM), corn germ meal (CGM), and sesame meal (SSM) were all purchased from Cargill Purina Company (Harbin, China). The MTM was procured from Daxinganling Liwokan Biotechnology Co., Ltd. (Daxinganling, China). The composition of the dry matter (DM), crude protein (CP), crude fat (EE), and crude ash were determined following the AOAC methods 930.15, 976.05, 920.39, and 942.05, respectively [24]. The starch content, neutral detergent fiber (NDF), acid detergent fiber (ADF), acid detergent lignin (ADL), neutral detergent insoluble protein (NDICP), acid detergent insoluble protein (ADICP), soluble crude protein (SCP), and non-protein nitrogen (NPN) contents were also analyzed following the methods of Van Soest [25] and Licitra [26]. The non-fibrous carbohydrate (NFC) content was calculated as being approximately 50 g per feedstuff, and all samples were oven-dried at 55 °C for 48 h, ground, sieved through a 1-mm mesh, and then stored at 4 °C for evaluating the chemical composition. The samples (*n* = 6) were collected per feedstuff and used as replicates.

(1) NFC(g/kgDM)=1000−NDF(g/kgDM)−CP(g/kgDM)−EE(g/kgDM)−Ash(g/kgDM)

The feedstuff AA profile was evaluated at Heilongjiang Academy of Agricultural Sciences (Harbin, China) using a fully automated amino acid detector (S-433D; Sekam Scientific Instruments Co., Ltd., Beijing, China), and 17 AA were assayed.

### 2.2. Protein and Carbohydrate Fractions

The nutritional value of the feedstuffs was evaluated using the CNCPS system following the method of Wang et al. [27]. The CNCPS divides the CP in the feed into non-protein nitrogen (PA), rapidly degradable protein (PB1), moderately degradable protein (PB2), slowly degradable protein (PB3), and unavailable protein (PC). As reported by Sniffen [28], the calculations were as follows:

(2) $$\begin{array}{l} PA(g/kgCP)=NFN(g/kgSCP)\times 0.001\times SCP(g/kgCP)\\ PB1(g/kg/CP)=SCP(g/kgCP)-PA(g/kgCP)\\ PB2(g/kgCP)=1000-PA(g/kgCP)-PB1(g/kgCP)-PB3(g/kgCP)-PC(g/kgCP)\\ PB3(g/kgCP)=NDIP(g/kgCP)-ADIP(g/kgCP)\\ PC(g/kgCP)=ADIP(g/kgCP) \end{array}$$

The carbohydrate was also divided into sugars and soluble fraction (CA), starch and pectin (CB1), fraction of available cell wall (CB2), and unavailable cell wall (CC), and was estimated as follows [28]:

(3) $$\begin{array}{l} CHO(g/kgDM)=1000-CP(g/kgDM)-EE(g/kgDM)-Ash(g/kgDM)\\ CC(g/kgCHO)=1000\times \left[NDF(g/kgDM)\times 0.001\times ADL(g/kgNDF)\times 2.4\right]/CHO(g/kgDM)\\ CB2(g/kgCHO)=1000\times \left[\begin{array}{l} NDF(g/kgDM)-NDIP(g/kgCP)\times 0.001\times CP(g/kgDM)\\ -NDF(g/kgDM)\times 0.001\times ADL(g/kgNDF)\times 2.4 \end{array}\right]/CHO(g/kgDM)\\ NSC(g/kgCHO)=1000-CB2(g/kgCHO)-CC(g/kgCHO)\\ CB1(g/kgCHO)=Starch(g/kgNSC)\times \left[1000-CB2(g/kgCHO)-CC(g/kgCHO)\right]\\ CA(g/kgCHO)=\left[1000-Starch(g/kgNSC)\right]\times \left[1000-CB2(g/kgCHO)-CC(g/kgCHO)\right]/1000 \end{array}$$

### 2.3. In Situ Rumen Degradation

Three fistulated Chinese Holstein cows with a body weight (BW) of 613 ± 10.2 kg (mean ± SD) in good health and body condition were utilized for in situ rumen degradation evaluation of the feedstuffs. The cows were fed daily at 06:00 and 18:00 and were offered fresh water without restriction. Table 1 shows the chemical and nutritional composition of the cows’ diets. Seven grams each of the feedstuffs was measured into pre-labelled nylon bags (*n* = 3) with a 50 μm pore diameter and size of 10 cm × 20 cm (Ankom Technology, Macedon, NY, USA), which were then tightened with rubber bands before being placed in a large mesh bag with a size 36 cm × 42 cm and then set for fermentation in the rumen ventral sac of each cow underneath the particulate mat layer for 0, 2, 4, 8, 12, and 16, 24, 36, and 48 h. After the fermentation process, the nylon bags were carefully washed with clean cold water, oven-dried at 55 °C for 48 h, and weighed. The residue was milled and sieved with a 1-mm sieve to analyze the DM, CP, and NDF. The rumen degradation rate and effective degradability were estimated following Ørskov and McDonald [29], as follows:

(4) $$p=a+b(1-e^{-ct})$$

(5) $$ED=a+b\left[c/(c+kp)\right]$$

where *p* is the degradation rate (%) at time *t* (h), *a* is the rapidly degradable fraction (%), *b* is the potentially degradable fraction (%), *c* is the rate of potentially degradable fraction, *ED* is the effective degradability, and the *kp* (outflow velocity) is 0.06 h^−1^ [30].

### 2.4. Intestinal Digestion

As described by Calsamiglia and Stern [31], the feedstuff small intestinal DM and protein digestibility were determined using the three-step in vitro method. A total of 1 g of pepsin (Sigma Chemical, St. Louis, MO, USA) was dissolved in pH 1.9 hydrochloric acid solution, and 1 g each of the residues from 16 h in situ rumen fermentation was pre-weighed into a nylon bag. The bags were then placed in the solution before being incubated at 39 °C for 1 h. The bags were then washed with cold water and placed in 1L pH 7.75 KH_2_PO_4_ buffer solution, which had been previously mixed with 50 µg thymol and 3 g trypsin (sigma P-7545, Sigma Chemical, St. Louis, MO, USA). The samples were then incubated again at 39 °C for 24 h before the bags were washed and dried at 55 °C for 48 h for N content determination. Intestinal digestion is calculated as follows:

Intestinal digestibility = [(Nutrient content of the residue after rumen degradation for 16 h − Nutrient content of the residue after digestion in the small intestine)/Nutrient content of the residue after rumen degradation for 16 h] × 100(6)

### 2.5. Molecular Structural Characteristics of Milk Thistle Protein Spectra 

Variations in the protein structure of the feedstuffs were determined by FTIR using a double-beam infrared spectrophotometer (WGH-30 Tianjin Port Dong Technology, Tianjin, China). A total of 2 mg each of the feed samples and 150 mg of kBr were used to prepare the disks, and scanning was carried out 128 times at a 4000~400 cm^−1^ range and 4 cm^−1^ spectral resolution. In total, five spectra were collected repeatedly per sample. The obtained spectra were analyzed with OMNIC 8.2 software (Thermo Nicolet Co., Madison, WI, USA). First, the spectrum was automatically smoothed, and the peak areas of the amide I and II bands were measured. Then, the peak height positions of the amide I and II bands, α-helix, and β-sheet were determined by Fourier automatic deconvolution function. 

### 2.6. Statistical Analysis

All data were sorted and organized in Excel (2016). Rumen degradation and intestinal digestibility data were analyzed on SAS software (version 9.4, SAS Institute Inc., Cary, NC, USA). The NLIN program was used to determine the degradation parameters a, b, and c values before being analyzed with other indicators. All other experimental data were also analyzed on SAS 9.4 software using the general linear model (GLM) procedure to perform variance analysis, and multiple comparisons were performed using the Tukey test, and the result was declared significant when *p* < 0.05.

## 3. Results and Discussion

### 3.1. Chemical Composition

Accessing readily available quality protein feed resources—which are not only affordable, but can also meet the nutrient requirement for animals—remains a challenge within the livestock industry. Over the years, feedstuffs like soybean, canola, and cottonseed meal have remained the major protein feedstuffs with great value. However, going by the recent rise in the market prices of conventional feedstuffs, due to high competition for its utilization between humans and animals, it is imperative to develop affordable, locally sourced protein alternatives. To achieve this, evaluating the basic nutritional components of potential alternative feeds is crucial for identifying their deficiencies as a basis for improving them for utilization as a protein feed resource for livestock. Studies have reported the nutritional composition of milk thistle seed cake or oil [32,33]. Still, to our knowledge, this is the first study to assess the nutritional and feeding value of MTM fully. The chemical composition of all of the evaluated feedstuffs is shown in Table 2. By comparison, we found that MTM has a higher NDF content than soybean meal, which had the lowest NDF of all of the feedstuffs (59% vs. 29%). The NDF composition of MTM was, in fact, not significantly different from that of corn germ and palm kernel meal. Its ADF and ADL compositions were also higher than those of the other feedstuffs. Its ash content was not significantly different from SBM, CNM, and CSM, while its starch, EE, and NFC content was lower than those of most other feedstuffs, including SBM.

NDF is vital for the digestive process of dairy cows as it has a significant impact on their feed intake, rumen fermentation, and nutrient utilization, and it is essential for maintaining the rumen environment and sustaining milk production and fat content [34,35]. When formulating the diets for high-yielding dairy cows, it is necessary to ensure an appropriate concentration of NDF while meeting energy density requirements, as this can maximize milk production and fat content [34]. According to the National Research Council (NRC) recommendations, they should have an NDF level of about 30–40% in their diet [36]. The result of this study revealed that MTM has a very high fiber content. Shi [35] reported that, during peak lactation, cows fed a high NDF diet had lower milk production, particularly the total milk yield, milk lactose and protein yield, the percentage of milk protein, and nitrogen efficiency. Moreover, even though ruminants need to consume sufficient NDF to ensure various physiological functions, they tend to obtain it from fresh and tender forage. The high lignin content in MTM, reaching up to 16.83%, may also negatively impact the digestion rate of the feed. NDF includes cellulose, hemicellulose, and lignin. For ruminant animals, the plant cellulose and hemicellulose can be fermented by rumen microorganisms and maximized as energy sources. However, lignin is less digestible and can impact the rumen digestion rate because it is not degraded in the rumen anaerobic environment but only aerobically; therefore, its level in feed has a negative correlation to DM digestibility [37,38]. NFC includes starch, sugars, and pectin, and it has been noted that increasing their levels in the diet can enhance milk production performance and benefit the use of energy and nitrogen in dairy cows [39]. From this study, MTM had an NFC composition of about 7%, which was relatively lower than that of soybean meal (16.67%); therefore, caution is needed when replacing it in ruminant ration. 

Furthermore, the CP content of MTM was lower (25%) compared to that of SBM, CNM, CSM, and SSM, but it was still higher than that of PKM, RBM, and CGM, making it a potential protein feed candidate with prospect. Its NDICP content was also lesser than that of other feedstuff, except for CNM, CSM, and RBM, while its ADICP content was higher than other feedstuffs, except for SSM. SCP and NPN are high-quality sources of rumen-degradable protein (RDP). MTM has relatively high levels of SCP and NPN, similar to SBM and RSM, indicating a higher proportion of rapidly degradable components in the rumen. However, it also has a high level of ADICP, which is less available to the rumen as an AA, reducing its feeding value [40]. The literature has revealed that, after pressing or extracting oilseed meals, only a small amount of fat is retained, yet there could be variations due to differences in extraction processes. In this study, PKM retained a higher fat content, and MTM had the lowest fat content among the feedstuff, at only 0.20%, indicating that it could be limited in essential fatty acids, such as linoleic and linolenic acid. A low fat content could also reduce its palatability and, consequently, the feed intake and growth performance of the animal. 

### 3.2. Amino Acid Profile 

The AA profile of MTM and other feedstuff is presented in Table 3. MTM has a poor AA composition, with leucine, histidine, arginine, and proline being all significantly lower than in the other feedstuffs. In addition, the proportions of aspartic acid, glycine, isoleucine, tyrosine, and total AA were lower than the other feedstuffs, except CGM. Its lysine proportion was also lower than that of SBM, CNM, and CSM, although still higher than that found in PKM, RBM, CGM, and SSM. Nevertheless, it is noteworthy that it has high methionine content, exceeded by only SSM, and it has high serine and cystine contents, exceeded by only SBM. The main limiting AAs for dairy cows are methionine, histidine, and lysine [41]. Even though MTM has a higher methionine content than SBM, it is lower in lysine and histidine composition; therefore, when used as a substitute for soybean meal, careful consideration is needed. Zhu previously reported on the protein and AA composition of *Silybum marianum* meal and observed a 47% CP, which differs greatly from this study. This variation could result from plant variety, origin, and processing techniques, which will, in turn, influence the difference in the AA composition as well. Generally, SBM has a high total AA content and a balanced AA proportion, and MTM has a considerable gap in comparison. As a result, when reducing the amount of SBM in the dairy cow ration, MTM may not have an advantage in terms of AA content, more so because its CP content is slightly lower. However, considering the price advantage, it is still an unconventional feed that is worth developing.

### 3.3. Protein and Carbohydrate Fraction 

Table 4 shows the CNCPS components of all of the feedstuffs. Regarding the protein fractions, MTM had a higher PA component than SBM, CNM, CSM, PKM, RBM, and CGM. The PA component is mainly related to NPN. Based on this study, MTM contains 4.34% NPN (Table 2), resulting in a PA component of 16.83%. NPN is made up specifically of the nitrogen present in the nucleic acids and ammonia, and it can be rapidly utilized by the rumen microorganisms to synthesize microbial protein [42], which provides most of the protein available in ruminants’ small intestine and constitutes about 50–80% of the total protein that is absorbed [43]. The PB component represents the true protein portion of the feed. Our results revealed that the PB1 component of MTM was similar to that of SBM, CNM, CSM, RBM, CGM, and SSM; moreover, its PB2 component was also not different (*p* > 0.05) from SBM, RB, and SS, and its PB3 component was lower than that of SBM, PKM, RBM, CGM, and SSM. PB1 and PB2 are rapidly and moderately degradable, respectively. The PB2 component is soluble in a neutral detergent but insoluble in a buffer. Accelerated degradation of feed protein could result in peptides and AA accumulation two hours after feeding [20]. It could be lost as microbial protein or result in increased ammonia production, because true protein is initially degraded into peptides and AA and later deaminated into ammonia nitrogen [44]. Therefore, regulating the feed content of PA and PB feeds can better control the rate of microbial protein, reduce nitrogen loss, and promote nitrogen utilization in feeds. MTM has optimum levels of these two components, similar to soybean meal, indicating that it has good protein quality. However, this study revealed that it had the highest PC component of about 10%, which may affect the evaluation of its protein composition because the PC represents protein bound to substances like lignin and tannins that rumen microorganisms cannot utilize [19]. 

Regarding the carbohydrate components, MTM has a significantly lower CA component than that of SBM, CNM, CSM, RBM, and SSM; moreover, its CB1 component was lower than that of SBM, RBM, and CGM. Its CB2 component was lower than that of CSM, PKM, and CGM, and not different from SBM, CNM, RBM, and SSM. It had the highest CC component, reaching about 61%. On a dry matter basis, its CHO content was significantly lower than PKM, RBM, and CGM, but higher than that of SBM, CNM, CSM, and SSM, and the NSC content in its CHO was lower than that of SBM. The CA component is the water-soluble fraction that is rapidly fermentable and is composed mainly of sugars and some proportions of oligosaccharides and organic acids [20], and the CB1 component is the fraction composed of starch and pectin. The MTM fractions of CA and CB1 are much lower than those of most protein feedstuffs. The CC component, which is the indigestible fraction composed of unavailable cell wall, accounts for 60.94% of the entire carbon component, which could lead to a lower digestibility of the carbon component and, therefore, limit the application of MTM. In short, MTM has a good proportion of protein components but a poor proportion of carbon components. To further develop this feedstuff for a better feeding performance, the digestibility of the carbon components needs to be improved in order to maximize its feeding value.

### 3.4. In Situ Rumen Degradation and Small Intestinal Digestibility 

The in situ DM, CP, and NDF rumen degradation is shown in Table 5. With respect to the rumen degradation kinetics of DM, the *a*-value of MTM was lower than that of SBM, CNM, RBM, and SSM, and the *b*-value was lower than that of the other feedstuffs. Similarly, it had the least effective degradability, the same as PKM. Among the CP rumen degradation kinetics, MTM had the highest *a*-value, and its *b*-value did not differ significantly from the others, except SSM, while its *c*-value was the lowest. Its CP effective degradation was not different from SBM and CNM and was higher than the others, while it had a rumen bypass protein lower than that of PKM, RBM, CGM, and SSM. Furthermore, it had the lowest NDF rapid degradation fraction, a slow degradation fraction lower than SBM and PKM, and an NDF effective degradation rate lower than CNM, CSM, PKM, CGM, and SSM.

The MTM rumen DM rapid, slow, and effective degradation rates were lower than those of most protein feeds determined in this experiment. This may be due to the excessively high NDF level and the relatively low rumen NDF effective degradation rate observed. The NDF content of MTM reached up to 59%, but the rumen NDF rapid, slow, and effective degradation rates were 0.17%, 30.41%, and 17.78%, respectively. The MTM low NDF degradation is also reflected and associated with the low DM degradation rate and relatively high CC component predicted by CNCPS. We speculate that this likely results from the excessively high ADL content in MTM, accounting for a large proportion of NDF, as ADL is negatively correlated with the rumen fiber digestion rate. Lignin is cross-linked with arabinoxylan, hindering the physical contact of hydrolytic microbial enzymes [45]. When MTM is used in large amounts in the ruminant diet, the low rumen digestion rate could cause rumen expansion or bloating by filling up a large space, hence reducing feed intake and making it challenging to meet the nutritional needs for high milk production [34]. Nevertheless, because of the good rumen CP effective degradation rate of MTM, which is similar to that of soybean meal, the nutritive value of the feedstuff can be further developed with a focus on lowering NDF and increasing the rumen DM and NDF degradation rate via processes such as microbial fermentation. 

A large portion of macronutrients, including lipids, escape starch, and proteins, are broken down and absorbed in the small intestine [2]. The small intestinal DM and RUP digestibility of MTM was lower than that of most of the other feedstuffs and far lower than that of SBM (Table 6). The high NDF composition of MTM can account for this, and the nutrient might have been bound to lignin and difficult to digest. The implication for dairy cows is less efficient nutrient uptake, which could impact their growth performance and milk production. Typically, a greater portion of the protein in a high-quality protein feedstuff should be available to reach the small intestine, where it is digested and absorbed [46]; therefore, MTM is not a good source of RUP. Nevertheless, its digestibility can be improved by the fermentation process, the addition of exogenous enzymes, or both [47].

### 3.5. Spectral Molecular Structure Characteristics of Milk Thistle Meal and Seven Conventional Protein Feedstuffs

The results in Table 7 reveal that the total area of amide I and II regions of MTM was significantly lower that of than other feedstuffs, except for RSM and CSM, while its peak area of amide II was lower than that of SBM and CSM but not different from the other feedstuff. In addition, its peak area ratio of amide I and II regions was higher than that of SBM, CNM, CSM, PKM, and CGM, while the peak height was higher than that of the other feedstuff. Additionally, the MTM protein secondary structure was significantly lower when compared to the other feedstuff, but the ratio of the α-helix to the β-sheet was not different. The peak height and area intensity of the amide I and II bands are related to the number of protein functional groups, and differences in their ratios signify variations in protein structural composition [48]. The amide I band was mainly composed of C=O stretching vibrations and a small amount of C-N stretching vibrations, while the amide II band was mainly composed of N-H bending vibrations and a small amount of C-N stretching vibrations [49]. In this study, SBM, CSM, CNM, and SSM exhibited higher values in the peak area and peak height of the amide I and amide II bands, possibly because of their higher total protein content. Conversely, PKM, RBM, CGM, and MTM showed lower values, due to their relatively lower protein content. 

The amide I band includes the secondary protein structures of α-helix and β-sheet spatial structures, both of which are related to protein digestion in animals [50]. The amide I to amide II band ratio and the α-helix to β-sheet ratio are often related to feed protein solubility, rumen degradation rate, and small intestinal digestibility [51]. MTM and RBM show no difference in the amide I to II bands ratio and α-helix to β-sheet height ratio, even though their rumen degradation rate and small intestinal digestibility do not exhibit similarity. Studies have shown that the ratio of the amide I band to the amide II band is closely influenced by deoiling methods [52] and feed types [53]. Although there is a correlation in the protein molecular structure between MTM and RBM, there are significant differences in their nutritional composition. Therefore, a spectral structure may not fully reflect the digestive characteristics of the feeds’ proteins.

## 4. Conclusions

Based on the outcome revealed in this study, milk thistle meal, in comparison to most other conventional feedstuffs, has a beneficial nutritional quality. Although it has a lower crude protein, it exhibits moderate levels of essential amino acids, especially methionine, has a significant fraction of indigestible protein and slowly digestible carbohydrates, and has high crude protein effective degradability and rumen undegradable protein values, making it a potential protein feed resource. However, caution is required when including milk thistle meal in the ruminant diet, because of its high fiber content, low effective degradability of dry matter, and neutral detergent fiber, coupled with a very low rumen-effective degradation rate of NDF. There is, therefore, a need to investigate further ways of improving its nutritional value and developing it as an unconventional feed suitable for dairy cattle.

## Figures and Tables

**Table 1:** Experimental diet composition and nutrient level (DM basis) %.

Items	Content
Ingredients	
Chinese wildrye	42.69
Corn silage	15.77
Corn	13.27
Wheat bran	3.75
Molasses	0.99
Soybean meal	3.18
Dried distillers’ grain	5.38
Cottonseed meal	2.08
Corn fiber feed	7.45
Corn germ meal	4.96
Premix ^1^	0.50
Nutrient composition ^2^	
NE_L_/(Mcal/kg)	1.30
CP	14.87
NDF	50.81
ADF	31.60
Ca	0.62
P	0.41

^1^ Each kilogram of premix contains VA 8,000,000 IU, VD 700,000 IU, VE 10,000 IU, Fe 1600 mg, Cu 1500 mg, Zn 10,000 mg, Mn 3500 mg, Se 80 mg, I 120 mg, and Co 50 mg. ^2^ Net lactation energy was calculated, and the rest was measured.

**Table 2:** Chemical composition of milk thistle meal and seven conventional protein feedstuffs (DM basis) %.

Items	Soybean Meal	Canola Meal	Cottonseed Meal	Palm Kernel Meal	Rice Bran Meal	Corn Germ Meal	Sesame Meal	Milk Thistle Meal	SEM	*p*-Value
DM	88.77 ^d^	90.78 ^a^	90.43 ^ab^	90.09 ^bc^	88.63 ^d^	89.60 ^c^	88.77 ^d^	89.62 ^c^	0.148	<0.0001
Ash	7.31 ^cd^	6.94 ^cd^	6.62 ^d^	4.72 ^e^	11.27 ^b^	1.25 ^f^	19.50 ^a^	7.97 ^c^	0.248	<0.0001
EE	2.29 ^cd^	3.29 ^b^	2.80 ^bcd^	7.86 ^a^	2.03 ^d^	2.92 ^bc^	2.34 ^cd^	0.20 ^e^	0.179	<0.0001
CP	44.54 ^c^	40.28 ^d^	48.16 ^a^	16.42 ^g^	16.72 ^g^	20.90 ^f^	46.09 ^b^	25.57 ^e^	0.328	<0.0001
SP	8.40 ^b^	9.01 ^b^	4.76 ^d^	0.39 ^e^	3.42 ^d^	4.32 ^d^	10.65 ^a^	6.74 ^c^	0.348	<0.0001
NDICP	12.71 ^b^	5.62 ^e^	5.71 ^e^	10.23 ^c^	5.57 ^e^	8.26 ^d^	15.36 ^a^	6.13 ^e^	0.416	<0.0001
ADICP	0.58 ^e^	1.95 ^c^	1.02 ^d^	1.31 ^d^	0.46 ^e^	0.37 ^e^	4.01 ^a^	2.61 ^b^	0.0759	<0.0001
NPN	0.59 ^d^	4.98 ^b^	2.84 ^c^	0.00 ^d^	2.20 ^c^	2.49 ^c^	8.61 ^a^	4.34 ^b^	0.258	<0.0001
NDF	29.19 ^b^	33.05 ^b^	35.57 ^b^	61.64 ^a^	31.69 ^b^	56.93 ^a^	35.72 ^b^	59.19 ^a^	1.564	<0.0001
ADF	8.90 ^f^	20.97 ^c^	17.88 ^cd^	34.27 ^b^	12.75 ^e^	15.11 ^ed^	17.92 ^cd^	43.15 ^a^	0.697	<0.0001
ADL	1.65 ^e^	8.17 ^bc^	7.00 ^c^	9.28 ^b^	4.53 ^d^	1.82 ^e^	7.10 ^c^	16.83 ^a^	0.294	<0.0001
Starch	2.29 ^c^	0.23 ^e^	0.22 ^e^	0.00 ^f^	6.51 ^b^	6.73 ^a^	0.00 ^f^	0.82 ^d^	0.0186	<0.0001
NFC	16.67 ^b^	16.46 ^b^	6.84 ^cd^	9.36 ^c^	38.29 ^a^	18.00 ^b^	2.47 ^d^	7.08 ^cd^	1.225	<0.0001

DM, dry matter; EE, ether extract; CP, crude protein; SCP, soluble crude protein; NPN, non-protein nitrogen; NDICP, neutral detergent insoluble crude protein; ADICP, acid detergent insoluble crude protein; NDF, neutral detergent fiber; ADF, acid detergent fiber; ADL, acid detergent lignin; NFC, non-fiber carbohydrate. ^a–g^ Values in the same line with different capital letter superscripts mean samples have significant differences (*p* < 0.001); SEM, standard error of the mean.

**Table 3:** Amino acid contents of milk thistle meal and seven conventional protein feedstuffs.

Items	Soybean Meal	Canola Meal	Cottonseed Meal	Palm Kernel Meal	Rice Bran Meal	Corn Germ Meal	Sesame Meal	Milk Thistle Meal	SEM	*p*-Value
Aspartate	5.45 ^a^	2.49 ^d^	4.36 ^b^	1.22 ^g^	1.33 ^f^	1.35 ^f^	2.59 ^c^	1.79 ^e^	0.017	<0.0001
Threonine	1.68 ^a^	0.70b ^bc^	1.01 ^b^	0.32 ^c^	0.39 ^c^	0.56 ^bc^	0.68 ^bc^	0.78 ^bc^	0.120	<0.0001
Serine	2.59 ^a^	0.56 ^d^	0.83 ^c^	0.28 ^f^	0.28 ^f^	0.42 ^e^	0.40 ^e^	1.12 ^b^	0.006	<0.0001
Glutamic acid	8.84 ^b^	6.66 ^d^	10.35 ^a^	3.21 ^f^	2.00 ^h^	2.90 ^g^	7.08 ^c^	3.93 ^e^	0.013	<0.0001
Glycine	2.07 ^a^	1.92 ^c^	2.02 ^b^	0.71 ^g^	0.80 ^f^	1.07 ^e^	1.87 ^d^	1.10 ^e^	0.008	<0.0001
Alanine	2.05 ^a^	1.61 ^d^	1.80 ^c^	0.63 ^h^	0.89 ^f^	1.21 ^e^	1.87 ^b^	0.82 ^g^	0.008	<0.0001
Cysteine	0.68 ^a^	0.31 ^d^	0.55 ^c^	0.11 ^g^	0.20 ^e^	0.14 ^f^	0.30 ^d^	0.61 ^b^	0.005	<0.0001
Valine	2.00 ^c^	2.01 ^c^	2.23 ^b^	1.02 ^f^	0.96 ^f^	1.35 ^d^	2.57 ^a^	1.22 ^e^	0.012	<0.0001
Methionine	0.62 ^d^	0.67 ^c^	0.61 ^d^	0.30 ^f^	0.15 ^g^	0.39 ^e^	1.71 ^a^	0.77 ^b^	0.005	<0.0001
Isoleucine	1.81 ^a^	1.50 ^c^	1.55 ^b^	0.59 ^g^	0.58 ^g^	0.75 ^f^	1.45 ^d^	0.93 ^e^	0.007	<0.0001
Leucine	2.92 ^a^	2.62 ^a^	2.80 ^a^	1.05 ^c^	1.07 ^c^	1.71 ^b^	2.58 ^a^	1.39 ^b^	0.007	<0.0001
Tyrosine	1.43 ^a^	0.97 ^d^	1.20 ^c^	0.37 ^h^	0.43 ^g^	0.56 ^f^	1.39 ^b^	0.72 ^e^	0.007	<0.0001
Phenylalanine	2.23 ^b^	1.53 ^c^	2.67 ^a^	0.77 ^e^	0.77 ^e^	0.93 ^d^	2.23 ^b^	0.90 ^d^	0.007	<0.0001
Lysine	2.66 ^a^	2.09 ^b^	2.03 ^c^	0.44 ^h^	0.72 ^f^	0.85 ^e^	0.59 ^g^	0.97 ^d^	0.008	<0.0001
Histidine	1.09 ^b^	1.01 ^c^	1.36 ^a^	0.28 ^h^	0.38 ^g^	0.66 ^e^	0.83 ^d^	0.44 ^f^	0.005	<0.0001
Arginine	3.18 ^b^	2.15 ^d^	5.71 ^a^	1.74 ^e^	1.02 ^h^	1.22 ^g^	2.51 ^c^	1.65 ^f^	0.009	<0.0001
Proline	2.41 ^a^	2.18 ^b^	1.80 ^d^	0.80 ^h^	0.92 ^g^	1.34 ^e^	1.99 ^c^	1.03 ^f^	0.006	<0.0001
Total Amino Acid	43.69 ^a^	30.98 ^d^	42.88 ^b^	13.84 ^g^	12.89 ^h^	17.41 ^f^	32.64 ^c^	20.16 ^e^	0.155	<0.0001

Values are expressed as a percentage of crude protein (%CP). ^a–h^ Values in the same line with different letter superscripts mean samples have significant differences (*p* < 0.001); SEM, standard error of the mean.

**Table 4:** CNCPS components of milk thistle meal and seven conventional protein feedstuffs.

Items	Soybean Meal	Canola Meal	Cottonseed Meal	Palm Kernel Meal	Rice Bran Meal	Corn Germ Meal	Sesame Meal	Milk Thistle Meal	SEM	*p*-Value
PA (%CP)	7.64 ^c^	12.35 ^b^	5.92 ^c^	0.00 ^d^	13.14 ^b^	11.96 ^b^	18.71 ^a^	16.83 ^a^	0.79	<0.0001
PB_1_ (%CP)	11.56 ^a^	10.02 ^ab^	3.95 ^ab^	2.34 ^b^	7.34 ^ab^	9.02 ^ab^	4.43 ^ab^	9.79 ^ab^	1.803	0.0071
PB_2_ (%CP)	54.54 ^bc^	63.72 ^b^	78.29 ^a^	35.35 ^e^	43.84 ^de^	39.46 ^de^	43.52 ^de^	49.28 ^cd^	2.276	<0.0001
PB_3_ (%CP)	23.97 ^d^	9.07 ^e^	9.72 ^e^	54.32 ^a^	32.90 ^c^	37.77 ^b^	24.63 ^d^	13.75 ^e^	1.060	<0.0001
PC (%CP)	2.30 ^d^	4.84 ^c^	2.13 ^d^	8.00 ^b^	2.78 ^d^	1.80 ^d^	8.71 ^b^	10.37 ^a^	0.265	<0.0001
CA (%CHO)	59.05 ^a^	44.10 ^abc^	29.04 ^cd^	27.60 ^cd^	53.39 ^ab^	26.05 ^d^	36.19 ^bcd^	18.68 ^d^	3.859	<0.0001
CB_1_ (%CHO)	5.00 ^c^	0.46 ^e^	0.51 ^e^	0.00 ^f^	9.30 ^a^	8.98 ^b^	0.00 ^f^	1.23 ^d^	0.0390	<0.0001
CB_2_ (%CHO)	27.33 ^cd^	15.77 ^de^	30.84 ^bc^	41.04 ^b^	21.76 ^cde^	59.14 ^a^	13.39 ^e^	19.14 ^cde^	2.739	<0.0001
CC (%CHO)	8.62 ^ef^	39.66 ^c^	39.61 ^c^	31.35 ^d^	15.55 ^e^	5.83 ^f^	53.08 ^b^	60.94 ^a^	1.589	<0.0001
CHO (%DM)	45.86 ^e^	49.50 ^d^	42.41 ^f^	70.99 ^b^	69.98 ^b^	74.93 ^a^	32.08 ^g^	66.27 ^c^	0.398	<0.0001
NSC (%CHO)	64.05 ^a^	44.56 ^b^	29.55 ^bc^	27.60 ^bc^	62.69 ^a^	35.03 ^bc^	36.19 ^bc^	19.92 ^c^	3.854	<0.0001

PA, non-protein nitrogen; PB1, rapidly degraded protein; PB2, intermediately degraded protein; PB3, slowly degraded protein; PC, unavailable protein; CHO, carbohydrate; NSC, non-structural carbohydrate; CA, sugars and soluble fraction; CB1, starch and pectin; CB2, fraction of available cell wall; CC, unavailable cell wall. ^a–g^ Values in the same line with different letter superscripts mean samples have significant differences (*p* < 0.001); SEM, standard error of the mean.

**Table 5:** Rumen degradation parameters of milk thistle meal and seven conventional protein feedstuffs.

Items	Soybean Meal	Canola Meal	Cottonseed Meal	Palm Kernel Meal	Rice Bran Meal	Corn Germ Meal	Sesame Meal	Milk Thistle Meal	SEM	*p*-Value
In situ dry matter (DM) rumen degradation kinetics										
a (g/kg)	357 ^a^	237 ^bc^	137 ^cd^	114 ^d^	321 ^ab^	136 ^cd^	281 ^ab^	132 ^d^	21.21	<0.0001
b (g/kg)	643 ^c^	682 ^bc^	636 ^cd^	886 ^a^	544 ^de^	764 ^b^	376 ^f^	464 ^ef^	19.03	<0.0001
c (g/kg h^−1^)	28.0 ^de^	37 ^cd^	41.1 ^cd^	16.2 ^e^	43 ^bc^	55 ^b^	89 ^a^	39 ^c^	3.06	<0.0001
ED (g/kg)	622 ^a^	571 ^bc^	510 ^d^	369 ^e^	602 ^ab^	577 ^bc^	541 ^cd^	364 ^e^	8.9	<0.0001
In situ crude protein (CP) rumen degradation kinetics										
a (g/kg)	202 ^b^	168 ^bc^	94.7 ^d^	119 ^d^	167 ^bc^	85 ^d^	212 ^b^	292 ^a^	13.01	<0.0001
b (g/kg)	798 ^a^	832 ^a^	852 ^a^	687 ^ab^	833 ^a^	902 ^a^	406 ^b^	708 ^ab^	66.20	0.0019
c (g/kg h^−1^)	26.9 ^b^	38.3 ^b^	40.5 ^b^	19.9 ^b^	23.6 ^b^	33.2 ^b^	95.4 ^a^	27.0 ^b^	7.01	<0.0001
ED (g/kg)	526 ^abc^	571 ^ab^	516 ^abc^	282 ^d^	473 ^c^	493 ^c^	496 ^bc^	577 ^a^	15.90	<0.0001
RUP (g/kg)	474 ^bcd^	429 ^cd^	484 ^bcd^	718 ^a^	527 ^b^	507 ^b^	504 ^bc^	423 ^d^	15.94	<0.0001
In situ neutral detergent fiber (NDF) rumen degradation kinetics										
a (g/kg)	249 ^a^	74.7 ^cd^	34.8 ^b^	5.16 ^cd^	6.83 ^cd^	7.5 ^cd^	8.86 ^c^	1.70 ^d^	18.3	<0.0001
b (g/kg)	657 ^a^	361 ^bc^	633 ^ab^	995 ^a^	85.10 ^bc^	311 ^bc^	55.21 ^c^	304 ^bc^	11.8	0.0005
c (g/kg h^−1^)	38.7 ^ab^	10.6 ^bc^	4.0 ^ab^	3.90 ^c^	30.8 ^abc^	26.7 ^abc^	39.8 ^ab^	56.2 ^a^	0.07	0.0005
ED (g/kg)	562 ^a^	33.13 ^f^	53.043 ^e^	80.12 ^d^	26.34 ^f^	123 ^c^	35.61 ^f^	177.8 ^b^	2.81	<0.0001

a, rapidly degradable fraction in rumen degradation; b, slowly degradable fraction in rumen degradation; a + b, potentially degradable fraction in rumen degradation; c, the degradation rate of the slowly degradable fraction; ED, effective degradability of the incubated samples; RUP, rumen undegradable protein. ^a–f^ Values in the same line with different letter superscripts mean samples have significant differences (*p* < 0.001); SEM, standard error of the mean.

**Table 6:** Small intestine digestibility of milk thistle meal and seven conventional protein feedstuffs.

Items	Small Intestinal Dry Matter Digestibility (g/kg)	Small Intestinal Protein Degradation Rate (g/kg)
Soybean meal	656 ^a^	895 ^a^
Canola meal	248 ^d^	483 ^c^
Cottonseed meal	366 ^b^	702 ^b^
Palm kernel meal	16.46 ^e^	559 ^c^
Rice bran meal	327 ^bc^	551 ^c^
Corn germ meal	95.5 ^f^	136 ^e^
Sesame meal	299 ^bc^	302 ^d^
Milk thistle meal	276 ^cd^	367 ^d^
SEM	14.88	23.15
*p*-Value	<0.0001	<0.0001

^a–f^ Values in the same line with different letter superscripts mean samples have significant differences (*p* < 0.001); SEM, standard error of the mean.

**Table 7:** Protein spectral molecular structure characteristics of milk thistle meal and seven conventional protein feedstuffs.

Items	Soybean Meal	Canola Meal	Cottonseed Meal	Palm Kernel Meal	Rice Bran Meal	Corn Germ Meal	Sesame Meal	Milk Thistle Meal	SEM	*p*-Value
Amide I										
A_Amide I and Amide II	14.27 ^b^	10.04 ^c^	9.29 ^c^	1.41 ^d^	3.50 ^d^	2.24 ^d^	19.45 ^a^	8.33 ^c^	0.858	<0.0001
A_Amide I	6.74 ^b^	5.36 ^b^	4.98 ^b^	0.35 ^d^	2.66 ^c^	1.77 ^cd^	10.55 ^a^	4.88 ^b^	0.461	<0.0001
A_Amide II	2.00 ^a^	1.36 ^ab^	1.94 ^a^	0.77 ^bc^	0.50 ^c^	0.51 ^c^	1.17 ^bc^	0.93 ^bc^	0.164	<0.0001
A_Amide I/Amide II ratio	3.62 ^c^	4.06 ^c^	2.59 ^d^	0.46 ^e^	5.33 ^b^	3.62 ^c^	9.04 ^a^	5.27 ^b^	0.200	<0.0001
Amide I and II										
H_Amide I	0.079 ^b^	0.075 ^b^	0.068 ^b^	0.019 ^d^	0.036 ^cd^	0.024 ^d^	0.11 ^a^	0.057 ^bc^	0.006	<0.0001
H_Amide II	0.036 ^a^	0.043 ^a^	0.043 ^a^	0.016 ^b^	0.012 ^b^	0.011 ^b^	0.049 ^a^	0.018 ^b^	0.003	<0.0001
H_Amide I/Amide II ratio	2.31 ^b^	1.74 ^cd^	1.62 ^de^	1.20 ^e^	3.13 ^a^	2.26 ^b^	2.18 ^bc^	3.10 ^a^	0.103	<0.0001
Protein secondary structure spectral structural characteristics										
H_α-helix	0.072 ^b^	0.072 ^b^	0.064 ^b^	0.018 ^d^	0.034 ^cd^	0.022 ^d^	0.011 ^a^	0.052 ^bc^	0.006	<0.0001
H_β-sheet	0.077 ^ab^	0.070 ^bc^	0.066 ^bc^	0.015 ^e^	0.031 ^de^	0.023 ^de^	0.103 ^a^	0.049 ^cd^	0.006	<0.0001
H_α-helix/β-sheet ratio	0.95 ^b^	1.03 ^b^	0.97 ^b^	1.30 ^a^	1.08 ^ab^	0.97 ^b^	1.03 ^b^	1.08 ^ab^	0.075	0.0526

A_Amide I and Amide II: peak area intensity of total amide I and amide II; A_Amide I: peak area intensity of Amide I; A_Amide I/Amide II ratio: peak area ratio of amide I to amide II; H_Amide I: peak height intensity of amide I; H_Amide II: peak height intensity of amide II; H_Amide I/Amide II ratio: peak height ratio of amide I to amide II; H_α-helix: peak height intensity of α-helix; H_β-sheet: peak height intensity of β-sheet; H_α-helix/β-sheet ratio: peak height ratio of α-helix to β-sheet. ^a–e^ Values in the same line with different letter superscripts mean samples have significant differences (*p* < 0.001); SEM, standard error of the mean.

## Data Availability

Data are contained within the article.
